# Mechanism of Modified Danggui Sini Decoction for Knee Osteoarthritis Based on Network Pharmacology and Molecular Docking

**DOI:** 10.1155/2021/6680637

**Published:** 2021-02-12

**Authors:** Chaoqun Feng, Min Zhao, Leiming Jiang, Ziang Hu, Xiaohong Fan

**Affiliations:** ^1^Department of Orthopedics, Hospital of Chengdu University of Traditional Chinese Medicine, Chengdu 610075, China; ^2^Chengdu University of Traditional Chinese Medicine, Chengdu 610075, China

## Abstract

**Objective:**

This study aimed to explore the mechanism of Modified Danggui Sini Decoction in the treatment of knee osteoarthritis via a combination of network pharmacology and molecular docking.

**Methods:**

The main chemical components and corresponding targets of Modified Danggui Sini Decoction were searched and screened in TCMSP database. The disease targets of knee osteoarthritis were summarized in GeneCards, OMIM, PharmGkb, TTD, and DrugBank databases. The visual interactive network of “drugs-active components-disease targets” was drawn by Cytoscape 3.8.1 software. The protein-protein interaction network was constructed by STRING database. Then, GO function and KEGG pathway enrichment were analyzed by Bioconductor/R, and the pathway of the highest degree of correlation with knee osteoarthritis was selected for specific analysis. Finally, molecular docking was used to screen and verify core genes by AutoDockTools software.

**Results:**

Seventy-one main components of Modified Danggui Sini Decoction and 116 potential therapeutic targets of knee osteoarthritis were selected. The KEGG pathway and the GO function enrichment analysis showed that the targets of Modified Danggui Sini Decoction in the treatment of knee osteoarthritis were mainly concentrated on PI3K-Akt signaling pathway, TNF signaling pathway, IL-17 signaling pathway, apoptosis signaling pathway, Toll-like receptor signaling pathway, Th17 cell differentiation signaling pathway, HIF-1 signaling pathway, and NF-*κ*B signaling pathway. It mainly involved inflammatory reaction, regulation of apoptotic signaling pathway, cellular response to regulation of inflammatory response, cellular response to oxidative stress, and other biological processes. The molecular docking results showed that ESR1-wogonin, MAPK1-quercetin, RELA-wogonin, RELA-baicalein, TP53-baicalein, TP53-quercetin, and RELA-quercetin have strong docking activities.

**Conclusion:**

Modified Danggui Sini Decoction has the hierarchical network characteristics of “multicomponent, multitarget, multifunction, and multipathway” in the treatment of knee osteoarthritis. It mainly regulates the proliferation and apoptosis of chondrocytes by regulating the PI3K-Akt signaling pathway and establishes cross-talk with many downstream inflammatory-related pathways to reduce the overall inflammatory response. Meanwhile, HIF-1 expression was used to ensure the normal function and metabolism of knee joint under hypoxia condition, and the above processes play a key role in the treatment of knee osteoarthritis.

## 1. Introduction

Knee osteoarthritis (KOA) is a common degenerative bone and joint disease, which is caused by the imbalance of degradation synthesis coupling of chondrocytes, extracellular matrix, and subchondral bone under the action of mechanical and biological factors [1]. Among them, articular cartilage damage is the most important pathological change of KOA, which is mainly mediated by inflammatory reactions, leading to chondrocyte apoptosis and cartilage matrix degradation [2].

Modified Danggui Sini Decoction is a classic prescription of Traditional Chinese Medicine (TCM) in treating KOA. It is composed of Danggui nourishing blood and Guizhi warming meridians, which is combined as the monarch medicine. Baishao helps Danggui to nourish blood and harmonize nutrient, and Xixin helps Guizhi to warm and dredge blood vessels. It is supplemented with Tongcao, Niuxi, and Duzhong to invigorate kidney and bone, leading meridians to the affected areas. The whole prescription is used for warming yang and dispersing cold, nourishing blood and unblocking pulse, warming without dryness, tonifying without stagnation, and playing the effect of “warming meridians, dispersing cold, nourishing blood, and unblocking pulse” [3]. Previous clinical studies have confirmed that this prescription is safe and effective in the treatment of KOA, but there is still a lack of relevant research on its mechanism [4].

Network pharmacology, based on the discipline concept of “multigene, multitarget, and multidisease,” coincides with the medication thinking of TCM and has become a new mode which is suitable for the systematic development of TCM. Based on the network pharmacology, the chemical components of Modified Danggui Sini Decoction were scientifically screened and systematically predicted, and the “drugs-active components-disease targets” interaction network was established. Combined with GO function analysis, KEGG pathway enrichment analysis, and molecular docking technology, the mechanism and scientific connotation of Modified Danggui Sini Decoction in the treatment of KOA were revealed, providing an objective experimental basis for clinical application of TCM treatment of KOA, and providing new ideas and methods for the treatment of orthopedic and traumatology related diseases guided by TCM theory.

## 2. Data and Methods

### 2.1. Chemical Components and Targets of Modified Danggui Sini Decoction

Choose TCMSP (https://tcmspw.com/) Database [5], with the oral bioavailability (OB) ≥30% and drug-likeness (DL) ≥0.18 as the screening conditions [6, 7], the constituent herbs of Modified Danggui Sini Decoction were searched in turn, and the main chemical components of the prescription were obtained after supplementing the common components recorded in *Chinese Pharmacopoeia* [8]. Meanwhile, the corresponding targets of the above chemical components were sorted out, and the target genes annotation was completed by selecting the species as “*Homo sapiens*” in UniProt (https://www.uniprot.org/) database.

### 2.2. Targets Genes of KOA

We selected GeneCards [9], OMIM [10], PharmGKB [11], TTD [12], and DrugBank [13] disease-related databases and searched with “knee osteoarthritis” as the keyword. Among them, the retrieval results of GeneCards database were filtered with “correlation score >1.” The related genes of KOA were collected and a Venn map was drawn.

### 2.3. Potential Targets of Modified Danggui Sini Decoction in the Treatment of KOA

Using R language to match the annotated compound targets and the summarized KOA disease targets, the intersection genes were derived, and the potential targets of Modified Danggui Sini Decoction in treating KOA were obtained, and a Venn diagram was drawn.

### 2.4. Construction of Regulatory Network and Protein-Protein Interaction Network

The software of Cytoscape 3.8.1 [14] was used for visual analysis, and the above chemical components and target relationship were imported into the software, and the regulatory network diagram of “drugs-active components-potential therapeutic targets of disease” was drawn, and the color and shape of the visualization grid were adjusted according to different node properties.

The obtained potential therapeutic targets were imported into the STRING (https://string-db.org/) network platform [15], the research species was set as “*Homo sapiens*,” the highest reliability (score >0.9) was selected by comprehensive scoring, the discrete targets were hidden, the network diagram of protein-protein interaction (PPI) was constructed, and TSV format file was exported. The TSV file was imported into the software of Cytoscape 3.8.1, and the topological analysis was carried out by CytoNCA plug-in. With the values of betweenness, closeness, degree, eigenvector, and local average connectivity-based method and network greater than the median value, the core gene network was obtained.

### 2.5. GO Function and KEGG Pathway Enrichment Analysis

Go function analysis and KEGG pathway enrichment analysis were performed using Bioconductor (https://www.bioconductor.org/) platform and R language to analyze the GO function and KEGG pathway enrichment of the potential targets for the treatment of KOA. Through the related scripts, the tables of GO function analysis and KEGG pathway enrichment analysis were derived by R language. In GO function analysis, the top 10 items of biological process (BP), cellular component (CC), and molecular function (MF) were selected for visualization. In KEGG pathway enrichment analysis, the top 30 items were selected for visualization. The barplot was used to draw the histogram, and the bubble was used to make the bubble diagram.

### 2.6. Molecular Docking

The core genes were selected to find the related drug components in the compound regulatory network as small molecular ligands. The 2D structure information of drug chemical components was downloaded from PubChem (https://www.ncbi.nlm.nih.gov/) platform and converted into the 3D structure by ChemBio3D software, and the energy optimization of MM2 was carried out to complete the preparation of small molecule ligands. The 3D structure of the candidate target proteins was downloaded from PDB (http://www.rcsb.org/) database, and then the protein receptors were prepared after the water molecules and ligands were removed by PyMOL2.4.0 software. Autodocktools software was used to read the receptor files, which were converted to PDBQT format after hydrotreating ion modification. The ligand files were also converted to PDBQT format for saving and then converted into the 2D structure to draw the active pockets. Finally, AutoDock vina software will be used for molecular docking, and the lowest free energy model is selected for visual analysis.

## 3. Results

### 3.1. Main Components of Modified Danggui Sini Decoction and Treatment Targets of KOA

Through TCMSP database, the constituent herbs of Modified Danggui Sini Decoction were searched in turn. After screening by “OB ≥30% and DL≥0.18”, we searched the *Chinese Pharmacopoeia* for supplement and got 71 main chemical components of Modified Danggui Sini Decoction, including 3 kinds of Danggui, 7 kinds of Guizhi, 13 kinds of Baishao, 8 kinds of Xixin, 4 kinds of Tongcao, 20 kinds of Niuxi, and 16 kinds of Duzhong (Table 1). A total of 931 target genes were obtained by UniProt gene annotation simultaneously.

**Figure 1:**
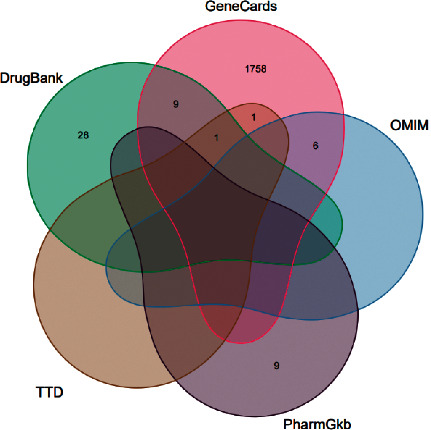
Venn diagram of KOA disease targets.

**Figure 2:**
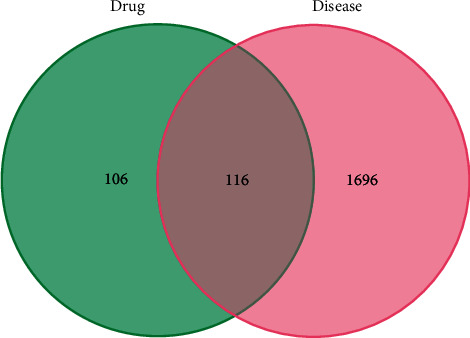
Venn diagram of Danggui Sini Decoction and KOA targets.

Through GeneCards, OMIM, and other disease-related databases, a total of 1812 KOA disease targets were collected (Figure 1). By matching the targets of Modified Danggui Sini Decoction with KOA related targets, one hundred and sixteen cross-genes were derived, which were potential targets of Modified Danggui Sini Decoction in treating KOA (Figure 2).

### 3.2. Construction of Regulatory Network and PPI Network

**Figure 3:**
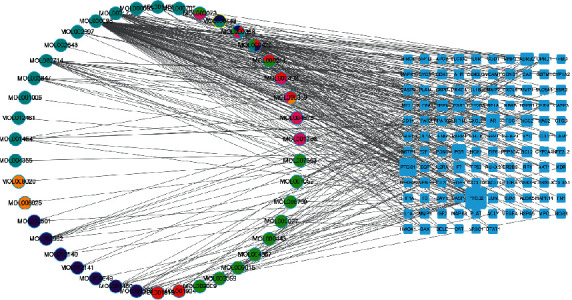
The “drug-active components-disease target” network. *Note.* the pink round is Guizhi, the green one is Duzhong, the red one is Baishao, the purple one is Xixin, the orange one is Tongcao, the cyan one is Niuxi, and the blue one is Danggui.

Using Cytoscape 3.8.1, the regulatory network of “drugs-active components-disease targets” was drawn (Figure 3). The network consists of 42 chemical component nodes, 116 potential therapeutic targets, and 306 edges. The circular node represents the chemical composition of the drug, and the rectangular node represents the gene target. The size of the visualization node is adjusted according to the degree value of the gene targets. The results showed that the top five components with the highest degree were quercetin (degree = 88), kaempferol (degree = 35), wogonin (degree = 29), baicalein (degree = 19), and beta-sitosterol (degree = 14). It is speculated that these components may be the key active components of Modified Danggui Sini Decoction in treating KOA.

**Figure 4:**
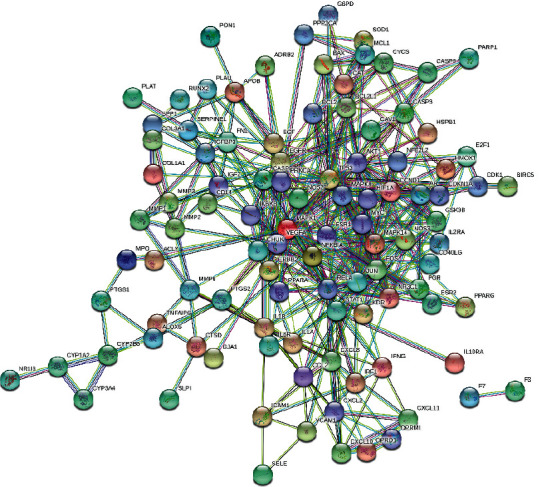
Protein-protein interaction network.

**Figure 5:**
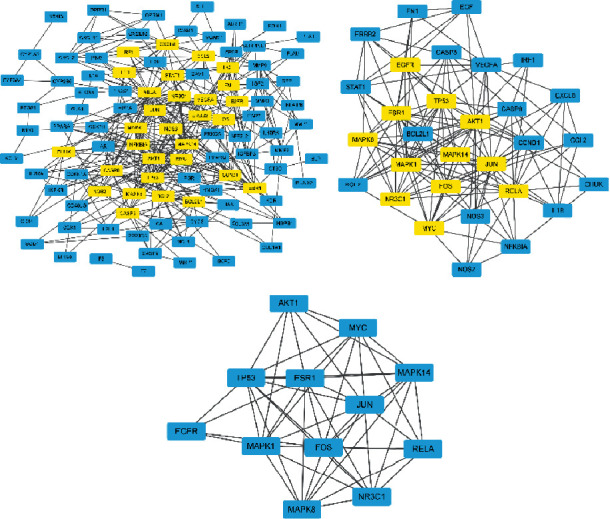
Core genes. *Note.* Yellow nodes are the core genes obtained after screening.

The potential therapeutic targets of Modified Danggui Sini Decoction in the treatment of KOA were imported into the STRING network platform to obtain PPI network diagram (Figure 4). The network contains 103 nodes and 401 edges. After calculating the median value of each parameter, thirty targets were obtained with “betweenness >46.20, closeness >0.21, degree >6, eigenvector >0.05, local average connectivity-based method >2.40, and network >3.10” as the first screening parameters. Similarly, twelve core targets were obtained by the second screening with parameters greater than median value, i.e., “betweenness >7.77, closeness >0.55, degree >8, eigenvector >0.13, local average connectivity-based method >4.14, and network >5.31” (Figure 5, Table 2).

### 3.3. GO Function and KEGG Pathway Enrichment Analysis

**Figure 6:**
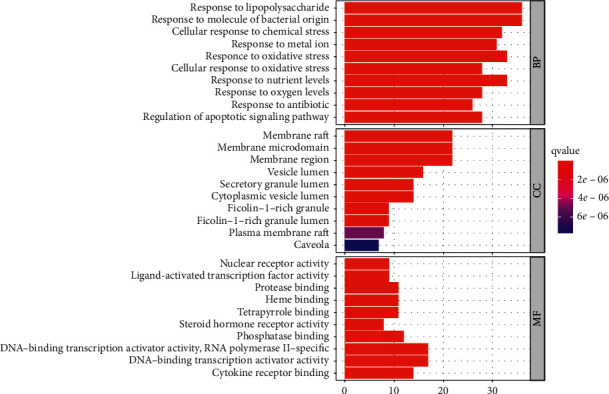
Go function analysis.

After GO function analysis, a total of 2363 GO entries were obtained (*P* < 0.05), and the top 10 items of BP, CC, and MF were selected for visualization (Figure 6). In the histogram, the redder the color is, the higher the enrichment degree is and the stronger the possibility of drug target is. According to the results of the biological processes, active components of Modified Danggui Sini Decoction in the human body mainly include response to lipopolysaccharide, response to molecular of bacterial origin, cellular response to chemical stress, response to metal ion, and response to oxidative stress. Cellular components mainly include membrane raft, membrane microdomain, membrane region, vesicle lumen, secretary granule lumen. The molecular functions mainly include nuclear receptor activity, ligand-activated transcription factor activity, protease binding, heme binding, and tetrapyrrole binding. The rich biological functions, to some extent, explain the reason why the same prescription can treat multiple diseases and also lay the foundation for exploring the effective ingredients and searching for signaling pathways.

**Figure 7:**
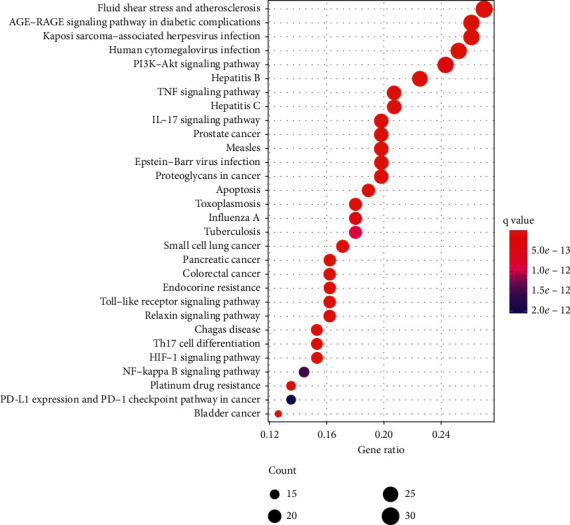
KEGG pathway analysis.

**Figure 8:**
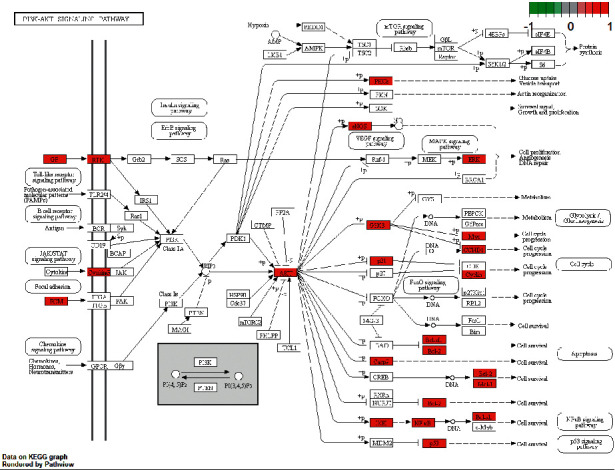
PI3K-Akt signaling pathway.

Through KEGG pathway enrichment analysis, a total of 150 related signal pathways of Modified Danggui Sini Decoction in the treatment of KOA were obtained (*P* < 0.05), and the top 30 items were listed for visual analysis (Figure 7). In the bubble diagram, the abscissa represents the ratio of the gene, the color also reflects the enrichment degree, and the bubble size represents the number of genes. The enrichment pathways mainly include fluid shear stress and atherosclerosis, AGE-RAGE signaling pathway in diabetic complications, kaposi sarcoma-associated herpesvirus infection, human cytomegalovirus infection, PI3K-Akt signaling pathway, hepatitis B, TNF signaling pathway, and IL-17 signaling pathway. The PI3K-Akt pathway, which is closely related to inflammatory response and bone metabolism, is selected as an example (Figure 8). The red labeled nodes are the targets of Modified Danggui Sini Decoction, which indicates that the prescription plays a key role in the PI3K-Akt signaling pathway by regulating marker targets.

### 3.4. Molecular Docking

**Figure 9:**
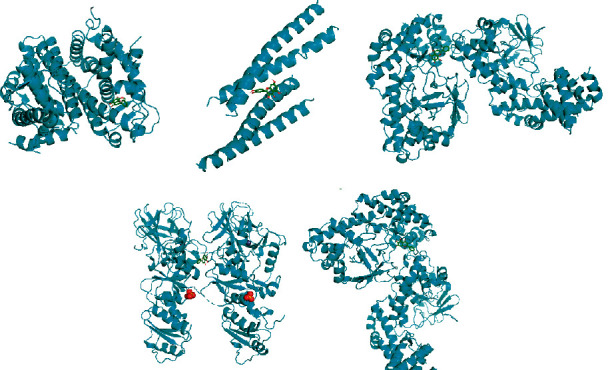
Molecular docking. *Note.* A is ESR1-wogonin molecular docking, B is MAPK1-quercetin molecular docking, C is RELA-wogonin molecular docking, D is RELA-baicalein molecular docking, and E is TP53-baicalein molecular docking.

In order to further verify the prediction results of the network, and to elaborate the mechanism and scientific connotation of Modified Danggui Sini Decoction as a classic Chinese medicine prescription in the treatment of KOA, quercetin, kaempferol, wogonin, baicalein, and *β*-sitosterol were selected as the top active components for molecular docking with Jun, TP53, ESR1, MAPK1, and RELA in turn. The binding energy between drug component ligands and target receptors is an important indicator to evaluate the binding capacity. It is generally considered that the docking affinity is stronger when the binding energy is less than −5.0 kcal/mol, and the docking activity is extremely strong when the binding energy is less than −7.0 kcal/mol [16]. From the docking results (Figure 9, Table 3), it was found that ESR1-wogonin, MAPK1-quercetin, RELA-wogonin, RELA-baicalein, TP53-baicalein, TP53-quercetin, and RELA-quercetin had the lower binding energies. In addition, quercetin and wogonin have the largest number of receptors as ligands. It is speculated that Modified Danggui Sini Decoction mainly participates in the treatment of KOA from the above molecular docking process.

## 4. Discussion

As a cultural treasure inherited for thousands of years, TCM has accumulated rich clinical experience, especially in the field of Traditional Chinese Medicine prescriptions. With the advancement of the modernization of Chinese herbal medicine, many studies have applied network pharmacology to explore the pharmacological mechanism of herbal medicine and prescription [17–19], and to carry out the research on the compatibility law and action mechanism of TCM, which also provides new ideas and methods for the scientific development and innovation of Chinese medicine. In order to systematically understand the mechanism of Modified Danggui Sini Decoction in the treatment of KOA, OB and DL were used as the important criteria for drug composition screening, a total of 71 main chemical components were obtained, and 116 potential therapeutic targets were obtained by matching the corresponding drug targets with the KOA disease targets. After further statistical mining, 12 core targets, 2363 GO functional enrichment items, and 150 KEGG related pathways were obtained, which explained that Modified Danggui Sini Decoction has the hierarchical network characteristics of “multicomponent, multitarget, multifunction, and multichannel” in the treatment of KOA.

The classical compatibility theory of TCM emphasizes that the four parts of monarch, minister, adjuvant, and messenger are combined to achieve synergy and minimize toxic and side effects integrally. Among them, the “messenger” mainly provides the effects of guiding the active ingredients to reach the target organs and harmonizing the actions of these agents [20, 21]. In addition, according to modern pharmacology and network biology, the drug mechanism of the TCM rules has been verified from a molecular/system level [7]. In this study, Dazao and Gancao, as the “messenger,” did not participate in the direct treatment of the disease. Therefore, in order to avoid bias or redundancy in the results, the two herbs were not included.

### 4.1. Potential Active Ingredients of Modified Danggui Sini Decoction in Treating KOA

In this study, the top five chemical components with the highest degree were quercetin, kaempferol, wogonin, baicalein, and *β*-sitosterol. At present, a considerable number of studies have proved that quercetin can significantly inhibit articular chondrocyte apoptosis and delay cartilage degeneration by reducing oxidative stress and endoplasmic reticulum stress, so as to achieve the purpose of treating KOA [22–24]. In addition, kaempferol has obvious anti-inflammatory and therapeutic effects on arthritis by inhibiting inflammatory factors such as IL-1B, NO, and PGE2 [25]. Wogonin can treat osteoarthritis by inhibiting oxidative stress, inflammation, and matrix degradation related to osteoarthritis, and regulating the redox activity of chondrocytes [26]. Baicalein can effectively inhibit the expression of inflammatory factors and slow down chondrocyte apoptosis and cartilage degradation in the treatment of osteoarthritis [27]. *β*-sitosterol is one of the main components of cell membrane and has an estrogen-like effect. It is known to be effective for hypercholesterolemia, heart disease, immune system regulation, and cancer prevention [28, 29], but there is no treatment research in KOA field.

Therefore, the main active components and efficacy of Modified Danggui Sini Decoction are similar to the results of network pharmacology analysis. Meanwhile, it is speculated that *β*-sitosterol has the value of research and development in anti-KOA treatment.

### 4.2. Mechanism Analysis of Modified Danggui Sini Decoction in Treating KOA

KEGG pathway and GO function enrichment analysis showed that the targets of Modified Danggui Sini Decoction in the treatment of KOA mainly concentrated on PI3K-Akt signaling pathway, TNF signaling pathway, IL-17 signaling pathway, apoptosis, Toll-like receptor signaling pathway, Th17 cell differentiation, HIF-1 signaling pathway, and NF-*κ*B signaling pathway. It mainly involves inflammatory reaction, regulation of apoptosis signaling pathway, cell response to chemical stress, cell response to oxidative stress, and other biological processes.

Among them, the PI3K-Akt signaling pathway is an important signal transduction pathway for regulating cell proliferation, differentiation, and apoptosis and promoting related tissue regeneration [30]. Akt can also activate specific downstream targets and interact with NF-*κ*B, mTOR, and P53 pathways. TNF in the tumor necrosis factor signaling pathway is an inflammatory mediator with many biological effects. TNF-*α* can induce the production of IL-6 and activate the protease that decomposes cartilage and synovium [31]. Activation of Toll-like receptor signaling pathway can release inflammatory factors such as IL and TNF, activate NF-*κ*B in its downstream signaling pathway, and induce apoptosis of articular chondrocytes [32]. As a hypoxia inducible factor, HIF-1 can induce the survival of hypoxic chondrocytes in hypoxic environment by maintaining the hypoxia balance state, which plays an important role in ensuring the normal physiological function and metabolism of knee joint cartilage [33]. In the NF-*κ*B signaling pathway, a variety of upstream signal factors, including TNF, can activate IKK, thus express downstream genes of NF-*κ*B, including MMP-9, P65, and P50, and then produce a cascade of inflammatory reactions and accelerate the decomposition and destruction of articular cartilage [34]. In addition, the IL-17 signaling pathway promotes the expression of inflammatory factors, which leads to the degradation of cartilage matrix. The Th17 signaling pathway can regulate osteoclast differentiation and promote bone resorption [35]. Thus, Modified Danggui Sini Decoction mainly regulates the proliferation and apoptosis of chondrocytes by regulating PI3K-Akt pathway and establishes cross-talk with many downstream inflammatory-related pathways to reduce the overall inflammatory responses. Meanwhile, HIF-1 expression was used to ensure the normal function and metabolism of knee joint under hypoxia condition, so as to play a role in the treatment of KOA.

At present, among the many regulatory pathways involved in KOA, the specific mechanism of some signaling pathways is still unclear, and there may be more complex interactions between the pathways, which jointly participate in the proliferation and apoptosis of chondrocytes, as well as the synthesis and degradation of extracellular matrix. Recent studies have found that the PI3K-Akt-mTOR signaling pathway also plays an important role in the pathogenesis of KOA. mTOR, as another serine/threonine protein kinase downstream of PI3K-Akt, is closely related to cell apoptosis, autophagy, cell survival, and structural reorganization [36, 37].

In conclusion, the main active components and efficacy of Modified Danggui Sini Decoction are similar to the results of network pharmacology analysis. Meanwhile, it also points out the direction for the follow-up study on the mechanism of Modified Danggui Sini Decoction in the treatment of KOA. This study is only searched through the databases. It did not take into account the clinical dosage, decocting conditions, and other factors brought by the impact. Thus, the next step requires further experimental verification and clinical research.

## Figures and Tables

**Table 1:** Main chemical components of Danggui Sini Decoction.

Drug	Meridian tropism	Number of compounds	Part of components
Danggui	Liver, heart, spleen	3	Ferulic acid, beta-sitosterol, stigmasterol
Guizhi	Heart, lung, bladder	7	Beta-sitosterol, taxifolin
Baishao	Liver, spleen	13	Paeoniflorin, beta-sitosterol, kaempferol
Xixin	Heart, lung, kidney	8	Cryptopine, kaempferol
Tongcao	Lung, stomach	4	Sitosterol, tetrapanoside B_qt
Niuxi	Liver, kidney	20	Wogonin, baicalein, kaempferol, quercetin
Duzhong	Liver, kidney	16	Pinoresinol diglucoside, beta-sitosterol

**Table 2:** Core genes.

Name	Betweenness	Closeness	Degree	Eigenvector	LAC	Network
ESR1	47.50	0.69	16	0.28	7	11.33
MAPK8	22.59	0.63	12	0.23	6	7.68
TP53	53.06	0.71	17	0.30	7.41	12.82
EGFR	14.99	0.57	10	0.16	4.60	6.52
MAPK14	42.57	0.66	14	0.25	5.86	8.35
NR3C1	9.50	0.60	10	0.21	6.20	7.68
MAPK1	61.51	0.69	16	0.28	6.50	10.31
FOS	14.57	0.63	12	0.24	7	8.39
JUN	92.05	0.74	19	0.31	7.16	15.30
MYC	14.59	0.63	12	0.24	6.83	8.99
AKT1	76.72	0.67	15	0.24	4.80	8.17
RELA	71.50	0.67	16	0.24	5.38	10.97

**Table 3:** Molecular docking (unit: kcal/mol).

Molecule name	JUN	TP53	ESR1	MAPK1	RELA
Quercetin	−6.6	−7.2	NA	−8.2	−7.2
Kaempferol	−6.4	NA	NA	NA	−7.1
Wogonin	−6.4	−6.9	−9.0	NA	−7.8
Baicalein	NA	−7.4	NA	NA	−7.6
Beta-sitosterol	−6.7	NA	NA	NA	NA

## Data Availability

The datasets used and/or analyzed during the current study are available from the corresponding author on reasonable request.

## Supplementary Materials

Supplementary Materials Supplementary Table 1: all chemical components of Modified Danggui Sini Decoction. Supplementary Table 2: the results of GO function enrichment analysis. Supplementary Table 3: the results of KEGG enrichment analysis.
