# Supplementary material for: Mechanism of Modified Danggui Sini Decoction for Knee Osteoarthritis Based on Network Pharmacology and Molecular Docking
Source: Evid Based Complement Alternat Med. 2021 Feb 12;2021:6680637. doi: 10.1155/2021/6680637 (PMC7895562; doi:10.1155/2021/6680637)
Supplement: Supplementary Materials — Supplementary Table 1: all chemical components of Modified Danggui Sini Decoction. Supplementary Table 2: the results of GO function enrichment analysis. Supplementary Table 3: the results of KEGG enrichment analysis. [file 6680637.f1.zip › 6680637.f1/Supplementary Table 1 All chemical components of Modified Danggui Sini Decoction.pdf]

Supplementary Table 1: All chemical components of Danggui Sini Decoction

Drug	Component	OB	DL
Danggui	beta-sitosterol	36.91	0.75
Danggui	Stigmasterol	43.83	0.76
Danggui	ferulic acid	54.97	0.06
Baishao	11alpha,12alpha-epoxy-3beta-23-dihydroxy-30-norolean-20-en-28,12beta-olide	64.77	0.38
Baishao	paeoniflorgenone	87.59	0.37
Baishao	(3S,5R,8R,9R,10S,14S)-3,17-dihydroxy-4,4,8,10,14-pentamethyl-2,3,5,6,7,9-hexahydro-1H-cyclopenta[a]phenanthrene-15,16-dione	43.56	0.53
Baishao	Lactiflorin	49.12	0.8
Baishao	paeoniflorin	53.87	0.79
Baishao	paeoniflorin_qt	68.18	0.4
Baishao	albiflorin_qt	66.64	0.33
Baishao	benzoyl paeoniflorin	31.27	0.75
Baishao	Mairin	55.38	0.78
Baishao	beta-sitosterol	36.91	0.75
Baishao	sitosterol	36.91	0.75
Baishao	kaempferol	41.88	0.24
Baishao	(+)-catechin	54.83	0.24
Duzhong	40957-99-1	57.2	0.62
Duzhong	Mairin	55.38	0.78
Duzhong	beta-sitosterol	36.91	0.75
Duzhong	kaempferol	41.88	0.24
Duzhong	olivil	62.23	0.41
Duzhong	Erythraline	49.18	0.55
Duzhong	Acanthoside B	43.35	0.77
Duzhong	AIDS214634	92.43	0.55
Duzhong	3-beta-Hydroxymethyllenetanshiquinone	32.16	0.41
Duzhong	ent-Epicatechin	48.96	0.24
Duzhong	Yangambin	57.53	0.81
Duzhong	Eucommin A	30.51	0.85
Duzhong	(+)-medioresinol	87.19	0.62
Duzhong	(-)-Tabernemontanine	58.67	0.61
Duzhong	Cyclopamine	55.42	0.82
Duzhong	pinoresinol diglucoside	3.07	0.34
Guizhi	(-)-taxifolin	60.51	0.27
Guizhi	beta-sitosterol	36.91	0.75
Guizhi	sitosterol	36.91	0.75
Guizhi	(+)-catechin	54.83	0.24
Guizhi	ent-Epicatechin	48.96	0.24
Guizhi	taxifolin	57.84	0.27
Guizhi	Peroxyergosterol	44.39	0.82
Niuxi	poriferasta-7,22E-dien-3beta-ol	42.98	0.76

Niuxi	28-norolean-17-en-3-ol	35.93	0.78
Niuxi	bidentatoside,ii_qt	31.76	0.59
Niuxi	Spinoside A	41.75	0.4
Niuxi	β-ecdysterone	44.23	0.82
Niuxi	berberine	36.86	0.78
Niuxi	coptisine	30.67	0.86
Niuxi	wogonin	30.68	0.23
Niuxi	delta 7-stigmastenol	37.42	0.75
Niuxi	baicalein	33.52	0.21
Niuxi	Baicalin	40.12	0.75
Niuxi	epiberberine	43.09	0.78
Niuxi	beta-sitosterol	36.91	0.75
Niuxi	Inophyllum E	38.81	0.85
Niuxi	kaempferol	41.88	0.24
Niuxi	Spinasterol	42.98	0.76
Niuxi	Stigmasterol	43.83	0.76
Niuxi	palmatine	64.6	0.65
Niuxi	beta-daucosterol_qt	36.91	0.75
Niuxi	quercetin	46.43	0.28
Tongcao	sitosterol	36.91	0.75
Tongcao	paryriogenin A	41.41	0.76
Tongcao	paryriogenin I	45.26	0.79
Tongcao	Tetrapanoside B_qt	40.93	0.79
Xixin	4,9-dimethoxy-1-vinyl-$b-carboline	65.3	0.19
Xixin	Caribine	37.06	0.83
Xixin	Cryptopin	78.74	0.72
Xixin	sesamin	56.55	0.83
Xixin	[(1S)-3-[(E)-but-2-enyl]-2-methyl-4-oxo-1-cyclopent-2-enyl] (1R,3R)-3-[(E)-3-methoxy-2-methyl-3-oxoprop-1-enyl]-2,2-dimethylcyclopropane-1-carboxylate	62.52	0.31
Xixin	(3S)-7-hydroxy-3-(2,3,4-trimethoxyphenyl)chroman-4-one	48.23	0.33
Xixin	kaempferol	41.88	0.24
Xixin	ZINC05223929	31.57	0.83
